# Discovery of the Role of Tick Salivary Glands in Enhancement of Virus Transmission—Beginning of an Exciting Story

**DOI:** 10.3390/pathogens12020334

**Published:** 2023-02-16

**Authors:** Pavlína Bartíková, Iveta Štibrániová, Mária Kazimírová

**Affiliations:** 1 Institute of Virology, Biomedical Research Center, Slovak Academy of Sciences, Dúbravská Cesta 9, 845 05 Bratislava, Slovakia; 2 Institute of Zoology, Slovak Academy of Sciences, Dúbravská Cesta 9, 845 06 Bratislava, Slovakia

**Keywords:** tick-borne viruses, saliva-assisted transmission, immunomodulation, skin

## Abstract

There is increasing evidence that arthropod-borne pathogens exploit saliva of their vectors during the transmission process to vertebrate hosts. Extensive research of the composition of tick saliva and its role in blood-feeding and transmission of pathogens started in the late 1980s and led to a number of discoveries on the composition and function of salivary molecules, some of which are associated with pathogen transmission. The study by Jones et al. published in 1989 can be ranked among the pioneer works in this field as it demonstrated for the first time the role of tick salivary glands in enhancement of transmission of a tick-borne virus. Thogoto virus was used in the model and subsequently similar results were obtained for tick-borne encephalitis virus. After a relatively silent period of almost 20 years, interest in tick–arbovirus–host interactions emerged again in the 2010s. However, no particular salivary molecule(s) enhancing virus transmission has (have) been identified to date. Intensive research in this field will certainly lead to new discoveries with future implications in the control of transmission of dangerous tick-borne viruses.

## 1. Introduction

The importance of salivary gland secretions in tick feeding was first outlined in detail by Kemp et al. [1]. Saliva of hematophagous arthropods including ticks contains a large number of various bioactive compounds which modulate vertebrate host defence reactions such as haemostasis, vasoconstriction, inflammation, immune responses, and wound healing at the attachment site in the host skin and facilitate blood-feeding of ectoparasites [2,3]. Arthropod-borne pathogens were shown to exploit saliva of their vectors during the transmission process to the vertebrate hosts and also take advantage of the modulated feeding site in the host skin for their establishment and dissemination in vertebrate hosts; for reviews, see [4,5,6]. Extensive research of the role of arthropod saliva in blood-feeding and transmission of pathogens started in the 1980s. The saliva composition and functions of salivary molecules of a number of haematophagous arthropods have been described, but only a few molecules could be associated directly with pathogen transmission.

Enhancement of the infectivity of a pathogen by arthropod salivary gland extracts (SGE) was first demonstrated in the sandfly *Lutzomyia longipalpis* and the protozoan parasite *Leishmania major* that causes cutaneous leishmaniasis [7]. In the same period, Jones et al. [8] demonstrated by using a laboratory model of guinea pigs, *Rhipicephalus appendiculatus* ticks and Thogoto virus (THOV) (*Orthomyxoviridae*) that uninfected ticks acquired the virus when they co-fed on the same host with infected ticks, while no viraemia could be detected in the host blood. Based on these observations, a novel mode of arthropod-borne virus transmission, non-viraemic transmission (NVT) was proposed. This localized spread of virus without viraemia is of particular interest as it appears to be much more efficient than classical viraemic transmission. Investigations into the NVT dynamics showed an increase in the percentage of nymphs which acquired the virus with duration of co-feeding, whereas NVT occurred even after physical separation of the infected and uninfected ticks [9]. “What is responsible for NVT?” was a question that began to be answered by the study “Enhancement of virus transmission by tick salivary glands” by Linda Jones and colleagues. Jones et al. [10] discovered that NVT could be enhanced by inoculating experimental guinea pigs with a mixture of THOV and *R. appendiculatus* SGE and suggested that the enhancement was not a result of a direct action of SGE on the virus, but was rather achieved by manipulation of the immune response of the host. This phenomenon was named “saliva-activated transmission” [11,12], later renamed “saliva-assisted transmission” (“SAT”) [13] which means an indirect promotion of pathogen transmission through manipulation of host defences by vector saliva molecules. In the early 1990s, NVT and SAT were also demonstrated, hand in hand, for an important pathogen, the tick-borne encephalitis virus (TBEV) (*Flaviviridae*) transmitted by *Ixodes ricinus* and *I. persulcatus* [14,15,16,17,18,19].

Since the beginning of the 21th century, research has been focused on exploration of the transmission mechanisms of tick-borne bacteria (mainly *Borrelia burgdorferi* s.l.), and an increasing number of saliva molecules that promote SAT, but also a few vector molecules that are exploited by the pathogens directly during transmission have been discovered; for reviews, see [20,21,22,23]. In contrast, research on tick–arbovirus–host interactions slowed down, apparently due to the fact that experiments involving majority of zoonotic arboviruses must be performed in strict conditions requiring biosafety levels 3 and 4. SAT factors promoting virus transmission have gained attention again since the 2010s, which has been reflected in increasing numbers of papers published on this topic. In a few review papers, knowledge on the known effects of tick saliva on transmission of tick-borne viruses has been updated [20,24,25,26]. However, no particular salivary molecules directly involved in enhancing/assisting virus transmission have been identified to date.

## 2. Discovery

**Figure 1:**
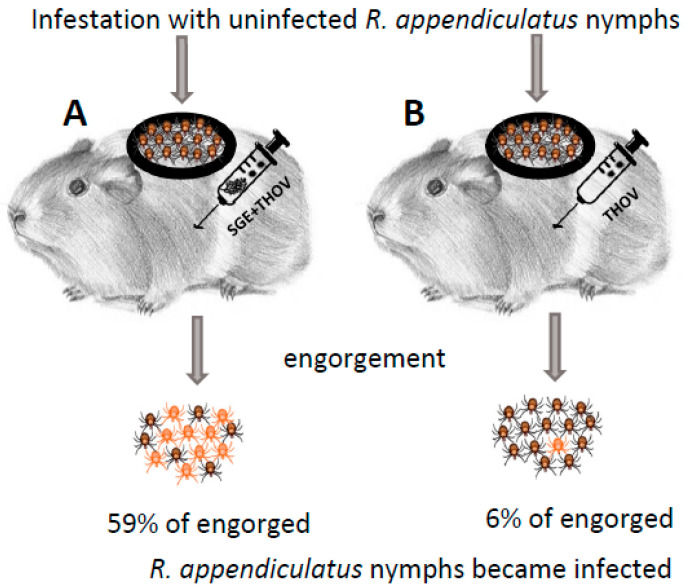
Experimental design in the publication by Jones et al. [10]. Each individual guinea pig in both experimental groups (**A**,**B**) was infested with 50 or 70 uninfected *R. appendiculatus* nymphs. Subsequently, guinea pigs from group A (7 animals) were injected with a mixture of salivary gland extracts (SGE) from uninfected *R. appendiculatus* or *A. variegatum* female ticks fed for 5 days and Thogoto virus (THOV, 5 × 10^3^ PFU), while guinea pigs in experimental group B (3 animals) were injected only with the same dose of THOV as in group A. Twelve days post engorgement, the presence of virus in individually homogenized nymphs was detected by plaque titration on Vero cells.

Jones et al. [8] demonstrated NVT of THOV from infected to uninfected *R. appendiculatus* ticks (natural vector of THOV) feeding simultaneously (co-feeding) on the same uninfected, non-viraemic guinea pigs. To further explore the mechanisms of NVT, Jones et al. [10] infested guinea pigs with uninfected *R. appendiculatus* nymphs (Figure 1). After infestation, the animals were inoculated subcutaneously in a distance of approximately 12 cm from the chamber containing feeding ticks with a mixture of THOV and SGE of uninfected 5-day fed adult *R. appendiculatus* or *Amblyomma variegatum* (another natural vector of THOV), extracts from *R. appendiculatus* ovaries, muscle, midgut, Malphigian tubules and whole ticks without salivary glands or mosquito SGE, or with THOV alone (control). The highest percentages of THOV-positive nymphs (59% and 58%, respectively) were detected among those that had fed on animals inoculated with a mixture of the virus and SGE of *R. appendiculatus* and *A. variegatum*. Further experiments were conducted to determine whether the enhancing factor(s) were present in *R. appendiculatus* salivary glands during different stages of feeding. For this purpose, similar to the previous experiment, guinea pigs were infested with *R. appendiculatus* nymphs and inoculated with a mixture of THOV and SGE derived from uninfected *R. appendiculatus* females at 0, 1, 4, 6 or 8 days of feeding. The highest percentage of infected nymphs (78%) was detected on guinea pigs that were inoculated with SGE of 6-day fed *R. appendiculatus* females, while enhancement of virus transmission was not observed when SGE of unfed or 1-day fed ticks was used. Another group of tick-infested guinea pigs was inoculated either with a mixture of SGE of 5-day fed *R. appendiculatus* and virus, or separately with SGE and virus into different parts of the skin. The authors observed enhanced transmission only in animals inoculated with the mixture of SGE and virus. No viraemia was detected in any of the experimental guinea pigs.

Three main results were demonstrated by the experiments performed by Jones et al. [10]: (1) specific enhancement of THOV transmission by tick SGE suggesting that tick salivary glands produce molecules that act at the tick–virus–host interface and facilitate NVT; (2) changes in the composition and/or concentration of the enhancing factor(s) in salivary glands during tick feeding; (3) localized effect of the enhanced virus transmission in the host skin.

## 3. Impact

The paper by Jones et al. [10] significantly impacted the research on the salivary glands and saliva (composition/function/identification of individual molecules) of several tick species in the context of tick–host interaction as well as the research on mutual interaction between viruses and ticks. According to Web of Science (https://www.webofscience.com/wos/woscc/basic-search (accessed on 30 November 2022)), this scientific work was cited in 103 published scientific articles in different scientific fields, mainly in Parasitology and Immunology.

**Figure 2:**
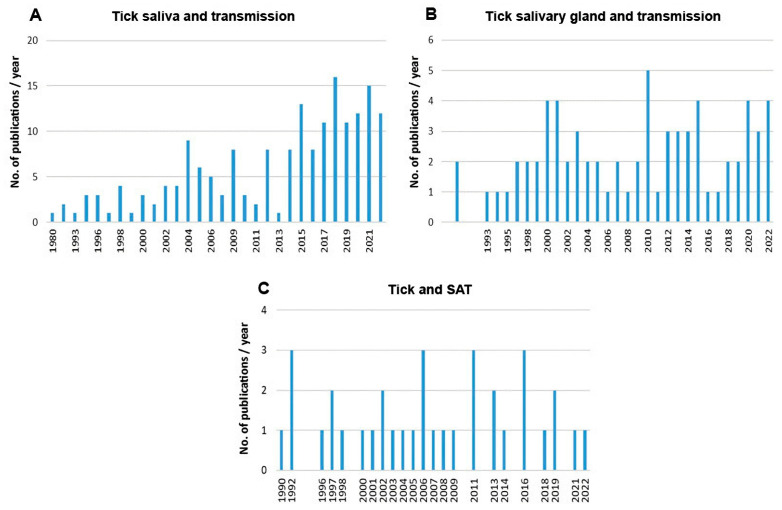
Summary of scientific publications (1989 till present) dealing with (**A**) tick saliva or (**B**) tick salivary gland and pathogen transmission and (**C**) tick and SAT.

**Figure 3:**
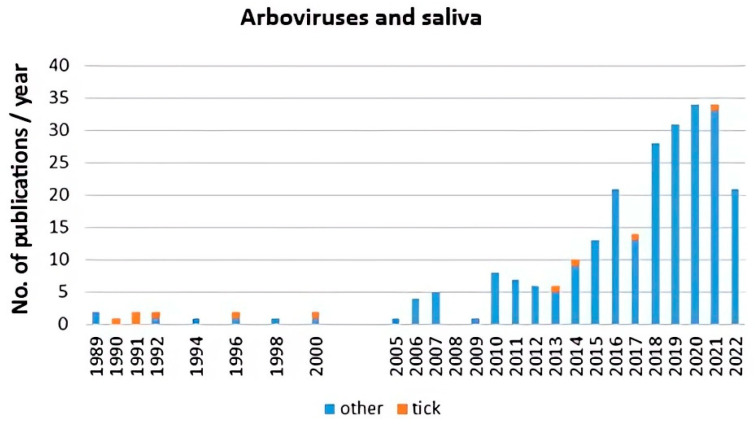
Scientometric analysis of interactions between arboviruses including tick-borne viruses and ticks (orange) and other arthropod saliva (blue) conducted on 267 publications obtained from PubMed (https://pubmed.ncbi.nlm.nih.gov; accessed on 30 November 2022) with key terms “arboviruses” and “saliva”.

**Figure 4:**
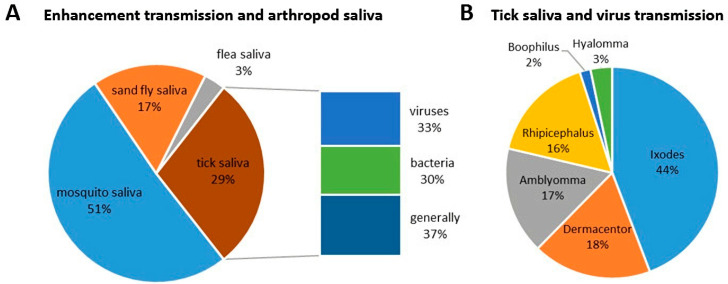
Proportional representation of published papers (1989–present) focused on (**A**) interactions between saliva from different blood-feeding arthropods, including ticks, and pathogen transmission and (**B**) between individual hard tick genera and transmission of tick-borne viruses.

Scientometric and bibliographic search on PubMed (https://pubmed.ncbi.nlm.nih.gov; accessed on 30 November 2022) using the terms “tick saliva” and “transmission”; “tick salivary gland” and “transmission”; “tick” and “SAT” (from 1989 till present) showed 179 scientific papers including 46 (25.7%) reviews (Figure 2A), 70 publications with 8 (11.4%) reviews (Figure 2B) and 35 publications including 6 (17.1%) reviews (Figure 2C), respectively. According to another bibliometric search on PubMed conducted with key terms “arboviruses” and “saliva”, 267 publications were obtained including 23 reviews (8.6%) and 10 (3.7%) studies on ticks (Figure 3). The growing trend in the number of publications underlines the importance of research of mutual interactions between arboviruses and arthropod vectors saliva. Focusing on individual arthropod vector groups, publications on “tick saliva” and “enhancement transmission” represented 29% of all publications and were second to mosquitoes; one third of these publications included tick-borne viruses (Figure 4A). Considering hard ticks, publications on species belonging to six genera were obtained; genus *Ixodes* spp. was the most studied (44%), followed by *Dermacentor* spp. (18%), *Amblyomma* spp., (17%), *Rhipicephalus* spp. (16%), *Hyalomma* spp. (3%) and *Rhipicephalus* (*Boophilus*) spp. (2%) (Figure 4B).

In the 1990s, follow-up studies to the work by Jones et al. [10] were carried out on different aspects of THOV transmission. Jones et al. [27] indicated that the SAT factors are probably proteins or peptides. It was further demonstrated that resistance of guinea pigs to repeated tick feeding decreased the rate of NVT [28], implying that host resistance to tick feeding may limit the spread of tick-borne viruses in nature. The effect of the SAT factor(s) on THOV was determined to be localized to the site of SGE inoculation and lasted at least three days, indicating the importance of modification of the tick attachment site in the host skin in mediating virus transmission [29]. Enhancement of THOV transmission was demonstrated only with SGE derived from metastriate tick species that are competent vectors of the virus, showing specificity of the SAT factor(s) [11]. In addition, the dynamics of SAT factor activities in mediating NVT was determined to differ in different phases of feeding of three-host and one-host metastriate tick species, indicating that SAT factor(s) are produced and secreted during feeding [30].

The THOV transmission model was adopted in subsequent studies and NVT and SAT were demonstrated for TBEV and its natural vectors *I. persulcatus* and *I. ricinus* (Prostriata) [14,15,19,31], as well as for *D. marginatus*, *D. reticulatus* and *R. appendiculatus* (Metastriata) [14,15,17,18,32]. Thus, in contrast to THOV, SAT for TBEV appeared to be less correlated with tick vector competence. Moreover, tick saliva was determined to also promote in vitro replication and production of the nucleocapsid viral protein of an insect-borne virus, which was demonstrated for *D. reticulatus* SGE and vesicular stomatitis virus [33,34,35].

Difference in the capacity to support co-feeding transmission of TBEV was observed between two natural hosts of immature *I. ricinus*, whereby significantly lower transmission rates were detected in ticks feeding on tick-resistant bank voles than on susceptible wild mice (*Apodemus* spp.) [16]. This difference was attributed, in part, to different speed of virus dissemination in the skin of mice and voles after attachment of infected ticks; i.e., dissemination of TBEV from the feeding site of infected *I. ricinus* to feeding sites of uninfected ticks was delayed in bank voles [36]. However, NVT was determined to occur in both naïve and virus immune wild rodents [37]. In TBEV-infected tick feeding sites in the skin of laboratory mice, the viral antigen was detected in migratory Langerhans cells and neutrophils, and migratory monocytes/macrophages produced infectious virus. These findings indicated that the tick feeding site is a focus of virus replication soon after virus transmission, and cells migrating from these sites probably act as vehicles for NVT [36]. However, the molecular mechanisms by which SAT factors mediate virus transmission in the host skin have not been revealed.

In addition to THOV and TBEV, indirect evidence of SAT has been documented for a few other RNA viruses (*Flaviviridae* and *Bunyavirales*) transmitted by hard ticks [38,39,40,41,42] and for African Swine Fever (ASF) virus (*Asfaviridae*), the only one DNA virus which is transmitted by soft ticks [43] (Table 1).

During the last two decades, several in vitro as well as in vivo studies aimed at revealing the mechanisms of immunomodulation at the tick–virus–host interface have been carried out. In vitro studies have focused on interactions of tick–host innate immune responses and revealed inhibition of the antiviral function of interferon [44] and suppression of natural killer (NK) cell functions [45,46,47,48] by tick SGE. Furthermore, *I. ricinus* saliva was determined to enhance TBEV replication in dendritic cells [49] and induce Akt pathway activation in TBEV-infected dendritic cells leading to decline in apoptosis and increase in cell viability [50]. Suppression of interferon responses in dendritic cells mediated by salivary cystatin (sialostatin L2) derived from *I. scapularis* was also observed [51]. Thus, sialostatin L2 could be considered as a potential SAT factor. Moreover, SGE of *I. scapularis* was determined to enhance the spread of Powassan virus (POWV) in the mouse brain when the animals were infected with a low viral dose [52]. All the reported effects can be considered as activities promoting arbovirus replication, transmission and dissemination. In contrast to *Flaviviridae*, no significant effects of *H. marginatum* SGE on response of dermal dendritic cells and Langerhans cells or on replication of Crimean-Congo haemorrhagic fever virus (*Bunyavirales)* were determined. SGE rather inhibited migration of antigen-presenting cells from the feeding site [53].

Application of modern high-throughput and systems biology approaches has greatly advanced the knowledge of tick sialotranscriptomes and proteomes. Different expression profiles for a number of genes in salivary glands, depending on the presence or absence of pathogenic microorganisms, have been revealed, and a few salivary molecules implicated in pathogen transmission have been identified [4]. Different salivary gland transcript expression profiles in response to duration of feeding and infection with Langat virus by *I. scapularis* nymphs were characterized [54]. In the same tick species, expression of salivary gland microRNAs during the earliest phase of POWV transmission to the vertebrate host differed, suggesting that the differently expressed microRNAs could regulate replication of the virus in host tissues [55]. In *I. ricinus* salivary glands infected with TBEV, differential expression of uncategorized genes, genes encoding proteases, Kunitz-type serine protease inhibitors, cytotoxins, and lipocalins during early stages of feeding was observed which may play an important role in virus transmission [56].

As indicated in earlier studies, SAT factors create immunologically favourable conditions in the tick attachment site that facilitate virus transmission and affect pathogenesis. In a series of in vivo studies, transcriptional analyses of early cutaneous responses of laboratory mice to tick attachment and transmission of flaviviruses were carried out. Attachment of uninfected *I. scapularis* nymphs elicited innate inflammatory response in the host skin [57,58]. In comparison with uninfected ticks, feeding of POWV-infected ticks recruited immune cells to the feeding site earlier [59] and macrophages and fibroblasts were identified as the early targets of virus infection at the tick feeding site [60]. By using RNA in situ hybridization, POWV was localized in dermal and hypodermal foci at the tick feeding site 24 h after attachment [61]. Transcriptional immunoprofile of early cutaneous immune responses of mice to feeding of TBEV-infected *I. ricinus* females was characterized by an inflammatory environment and a neutrophil-dominated immune response, highlighting the modulation of inflammatory chemokine and cytokine pathways in virus transmission. Mononuclear phagocytes and fibroblasts were identified as the primary targets for TBEV infection [62].

Recently, it was discovered that tick-borne Langat virus (LGTV) utilizes extracellular vesicles (exosomes) derived from *I. scapularis* ISE6 cell line for transmission of viral RNA and proteins to human keratinocytes and blood endothelial cells. The findings suggest that flaviviruses use arthropod-derived exosomes as a novel means for viral RNA and protein transmission from the vector to the host [63]. In vivo exosomes isolated from *A. maculatum* and *I. scapularis* saliva and salivary glands were shown to delay wound healing by modulating the levels of the chemokine CXXL12 and interleukin-8 in human skin keratinocytes [64]. Thus, exosomes are likely to facilitate tick feeding and pathogen transmission by modulating host skin immunity [65].

**Table 1:** Important milestones (in bold, with years in brackets) on the way towards identification of tick salivary molecules involved in transmission of tick-borne viruses.

Discovery of Non-Viraemic and Saliva-Assisted Transmission (1987, 1989)		
Thogoto virus	*Rhipicephalus appendiculatus*	[8,10]
	*Amblyomma variegatum*, *A. hebraeum*, *A. cajennense*, *Rhipicephalus evertsi*, *R. microplus*, *Hyalomma dromedarii*, *H. marginatum*	[11,30,66]
TBEV	*Ixodes persulcatus*	[18,19]
	*I. ricinus*, *Dermacentor reticulatus*, *D. marginatus*, *R. appendiculatus*	[14,15,16,17]
Louping ill virus	*I. ricinus*	[38]
Powassan virus	*I. scapularis*	[41]
CCHFV	*H. marginatum*	[39]
Palma, Bhanja	*R. appendiculatus*, *D. marginatus*	[40]
Heartland virus	*A. americanum*	[42]
African Swine Fever virus	*Ornithodoros porcinus*	[43]
**Discovery of the Effect of Host Resistance to Tick Infestation on Virus Transmission (1990)**		
Thogoto virus	*R. appendiculatus*	[28]
TBEV	*I. ricinus*	[16]
**Description of the Tick Feeding Site as a Focus for Virus Replication in the Early Phase of Transmission (1996)**		
TBEV	*I. ricinus*	[36]
**Modulation of Host Innate Responses by Tick Saliva or Salivary Gland Extracts (1994) and Enhancement of Virus Replication In Vitro (1998)**		
Natural killer cells	*I. ricinus*, *D. reticulatus*, *A. variegatum*, *Haemaphysalis inermis*, *H. concinna*	[45,46,47,48]
Interferon	*I. ricinus*, *D. reticulatus*	[44,47]
Dendritic cells	*I. ricinus*	[49,50]
Dendritic cells	*I. scapularis* salivary cystatin	[51]
Vesicular stomatitis virus	*D. reticulatus*	[33]
TBEV	*I. ricinus*	[49,50]
**Introduction of High-Throughput and Systems Biology Approaches**		
**A: Expression Profiles in Tick Salivary Glands During Feeding and Virus Infection (2012)**		
Langat virus	*I. scapularis*	[54]
Powassan virus	*I. scapularis*	[54,55]
TBEV	*I. ricinus*	[56]
**B: Immunoprofiling of Host Cutaneous Responses to Tick Attachment and Virus Infection (2012)**		
Powassan virus	*I. scapularis*	[58,59,60]
TBEV	*I. ricinus*	[62]
**Discovery of Extracellular Vesicles Serving as:**		
**A: Vehicles for Transmission of Viral RNA (2018)**		
Langat virus	*I. scapularis* cell line	[63]
**B: Modulators of Immune Responses in the Host Skin and Wound Healing (2020)**		
	*A. maculatum*, *I. scapularis*	[64]

To conclude, it is still not clear if tick-borne viruses exploit any specific salivary molecule(s) for transmission, or their transmission is promoted only indirectly by SAT factors, i.e., mainly by salivary molecules that modulate immune reactions in the host skin. In the second case, the SAT factors enhancing transmission of viruses may involve the same molecules as those which enhance transmission of tick-borne bacteria. However, there is a possibility that both mechanisms of transmission interact. Intensive research in this field will certainly lead to the discovery of new molecules with future implications in the control of transmission of dangerous tick-borne viruses.

## Data Availability

Not applicable.
